# H_1_-Antihistamine Up-Dosing in Chronic Spontaneous Urticaria: Patients' Perspective of Effectiveness and Side Effects – A Retrospective Survey Study

**DOI:** 10.1371/journal.pone.0023931

**Published:** 2011-09-01

**Authors:** Karsten Weller, Claudia Ziege, Petra Staubach, Knut Brockow, Frank Siebenhaar, Karoline Krause, Sabine Altrichter, Martin K. Church, Marcus Maurer

**Affiliations:** 1 Department of Dermatology and Allergy, Allergie-Centrum-Charité, Charité - Universitätsmedizin Berlin, Berlin, Germany; 2 Department of Dermatology, Universitätsmedizin Mainz, Mainz, Germany; 3 Department of Dermatology and Allergy Biederstein, Technische Universität, München, Germany; Ludwig-Maximilian-University, Germany

## Abstract

**Background:**

The guidelines recommend that first line treatment of chronic spontaneous urticaria should be second generation non-sedating H_1_-antihistamines with a positive recommendation against the use of old sedating first generation antihistamines. If standard dosing is not effective, increasing the dosage up to four-fold is recommended. The objective of this study was to obtain the chronic spontaneous urticaria-patient perspective on the effectiveness and unwanted effects of H_1_-antihistamines in standard and higher doses.

**Methodology/Principal Findings:**

This was a questionnaire based survey, initially completed by 368 individuals. 319 (248 female, 71 male, median age 42 years) had a physician-confirmed diagnosis of chronic spontaneous urticaria and were included in the results. Participants believed standard doses (manufacturers recommended dose) of second generation antihistamines to be significantly (*P*<0.005) more effective than first generation drugs. Furthermore, they believed that second generation drugs caused significantly (*P*<0.001) fewer unwanted effects and caused significantly (*P*<0.001) less sedation than first generation antihistamines. Three-quarters of the patients stated that they had up-dosed with antihistamines with 40%, 42% and 54% reporting significant added benefit from taking 2, 3 or 4 tablets daily respectively. The number of reports of unwanted effects and sedation following up-dosing were not significantly different from those reported for standard doses.

**Conclusions:**

This survey supports the urticaria guidelines recommendations that the first line treatment for chronic spontaneous urticaria should be second generation rather than first generation H_1_-antihistamines and that, if standard dosing is not effective, the dosage should be increased up to four-fold.

## Introduction

Chronic urticaria, defined as urticaria with episodes extending for more than 6 weeks [1], is a relatively common condition from which 0.5–1% of the population suffers at any single time [2] with all age groups and social strata affected. Furthermore, epidemiological studies have shown that this condition, which may last for months or even years [3], [4] may lead to major detrimental effects on quality of life, sleep deprivation and be associated with mental illness [5]–[7]. As a consequence, effective therapy is of paramount importance.

The guidelines issued following the third international consensus meeting on urticaria in 2008, a joint initiative of the Dermatology Section of the European Academy of Allergology and Clinical Immunology (EAACI), the EU-funded network of excellence, the Global Allergy and Asthma European Network (GA^2^LEN), the European Dermatology Forum (EDF) and the World Allergy Organization (WAO) [8], recommended that the first line treatment for chronic urticaria should be second generation, non-sedating H_1_-antihistamines. There is a positive recommendation against the routine use of old sedating first generation antihistamines. If standard dosing is not effective, increasing the dosage of non-sedating H_1_-antihistamines up to four-fold is recommended. Only if patients do not respond to this four-fold increase in dosage it is recommended that second-line therapies should be added to the antihistamine treatment. While many dermatologists use up-dosing regularly, the justification for it is based on long-standing clinical experience rather than good scientific evidence. It is only more recently that clinical trials have been performed to assess the response of H_1_-antihistamines at two times [9]–[12], three times [13], [14] and four times [15], [16] the licensed dose. However, what still remains to be done is to assess how patients view up-dosing with H_1_-antihistamines.

In this study, we have used a questionnaire to ask 319 patients with chronic spontaneous urticaria about the course of their condition, their previous treatment and its effectiveness with a special focus on their experience with up-dosing with H_1_-antihistamines. The results demonstrate that the patients rate second generation antihistamines to be more effective and better tolerated than first generation drugs and that many patients benefit from increasing the dose up to fourfold in case the standard licensed dose is not capable to adequately control their symptoms.

## Methods

### Objectives

The main objective of this study was to assess the effectiveness and side effects of sedating vs. non-sedating H_1_-antihistamines as well as of non-sedating H_1_-antihistamines in standard vs. higher than standard doses from the patients' perspective.

### Procedures and Participants

This was a questionnaire based retrospective survey initially completed by 368 individuals of whom 319 had a positive diagnosis of chronic spontaneous urticaria and were included in the results. Of these, 121 received questionnaires from their hospital or physician and 198 learnt about the survey on the internet from either the homepage of Allergie-Centrum-Charité or the web page of the Urticaria Network e.V. (www.urtikaria.net). The latter group completed the questionnaire online. Both physician administered and on-line questionnaires were identical. All questionnaires were completed anonymously and no Internet Protocol (IP) addresses were saved. Individuals from all 16 Federal States of Germany, 36% from rural areas, 38% from urban areas and 26% from metropolitan areas participated in the study. The only prerequisite for participation was that the individuals suffered from chronic spontaneous urticaria (recurrent spontaneous occuring wheals for >6 weeks) and were adults of age 18 or older. In addition, all participants were asked, if their urticaria was diagnosed by a physician (response options “yes” and “no”). Only those subjects who responded with “yes” were included in the analysis.

### Ethics

All participants received a patient information sheet before participation. To assure the full anonymity of the data set formal written informed consent was not obtained. However, the patient information contained a passage informing the participants that they declare their consent by completing the questionnaire. The survey and the indirect method of obtaining patient consent were both approved by the ethics committee (Ethikkommission der Charité - Universitätsmedizin Berlin, EA1/200/09) and the data protection commissioner of the Charité – Universitätsmedizin, Berlin.

### Data and Statistics

The data collected by this retrospective patient survey included the following: epidemiology and method of securing of the diagnosis, details of previous and current therapy, reasons for changing therapies, and experience with up-dosing with H_1_-antihistamines. In total, the questionnaire consisted of 54 questions, mostly involving Likert-scale ratings, quantitative questions, yes/no lists and multiple choice questions (the questionnaire can be provided upon request). The questions were assigned to the following subheadings: 1) general information (on the participants), 2) questions concerning the symptoms and course of disease, 3) questions concerning the diagnosis of urticaria, 4) questions concerning the treatment of urticaria, 5) questions concerning the impact of urticaria and 6) questions regarding care and the physician patient relationship. For the questions relevant to this manuscript, no time frame was specified in order to obtain as much patient experience as possible. Differences between first and second generation H_1_-antihistamines and standard and high dose therapy were tested using Fishers exact test.

## Results

### Patient details

The 319 respondents included in the survey (248 female and 71 male) of median age 42 years (range 18 to 76 years) stated that they had symptoms consistent with chronic spontaneous urticaria and had their diagnosis confirmed by a physician. Of the physicians who confirmed the diagnosis, 70% were dermatologists. The mean duration of the respondents' urticaria since diagnosis was 70±6 months (mean ± SEM) with wheals occurring daily in 48% of patients and several times a month in 95% of patients. Individual wheals lasted less than 24 hours in 87% of patients. Of the respondents, 25% rated their symptoms as moderate-to-severe, 46% as severe and 25% rated them as very severe. Furthermore, 68% reported that they also had symptoms of angioedema and 71% could not identify a cause for urticaria.

### Spectrum of H_1_-antihistamines used

**Figure 1:**
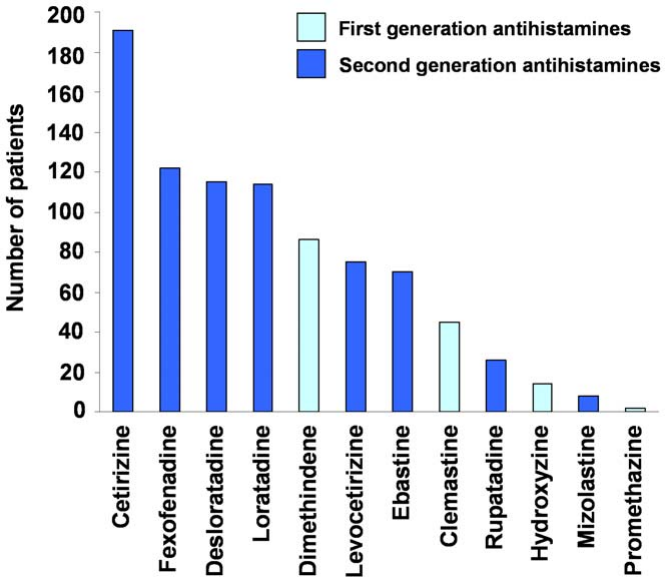
The spectrum of H_1_-antihistamines taken by the patients for the treatment of their chronic spontaneous urticaria. The results are expressed as the total number of participants who reported information on the efficacy of the itemized antihistamines in regular dose.

The patients in the survey stated that they had taken at least one of twelve H_1_-antihistamines from a given list, of which four, clemastine, dimethindene, hydroxyzine and promethazine, were first generation ‘sedating’ antihistamines and eight, cetirizine, desloratadine, ebastine, fexofenadine, levocetirizine, loratadine, mizolastine, rupatadine, were second generation, ‘non-sedating’ antihistamines (**Figure 1**).

Altogether, 205 respondents reported a total of 322 changes from one antihistamine to another during progression of their disease. The reason for changing medication was stated 230 times to be due to lack of effectiveness and 87 times due to side effects.

### First versus second generation H_1_-antihistamines

**Figure 2:**
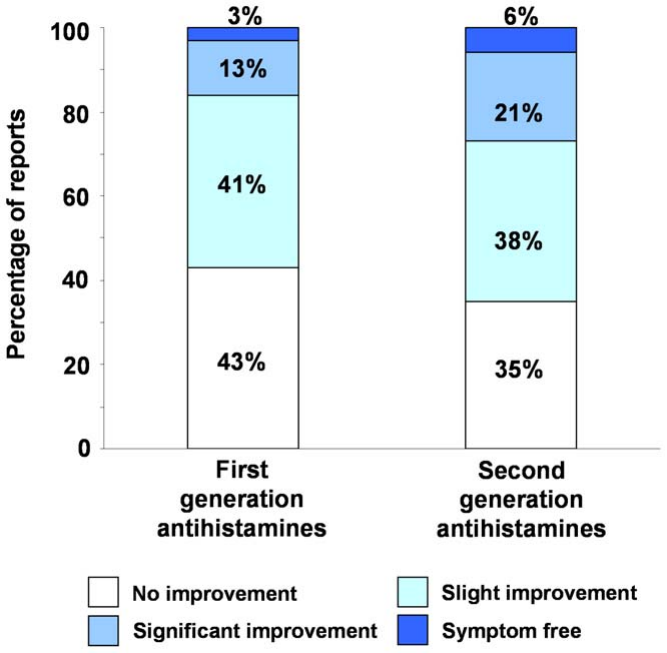
The comparative efficacy first and second generation H_1_-antihistamines in chronic spontaneous urticaria. The histograms were constructed from 147 and 721 patient reports respectively.

The patients' perspective of the comparative efficacy of first and second generation H_1_-antihistamines taken at the standard dose (manufacturers recommended or licensed dose) is shown in **Figure 2**. For statistical comparisons, the responses were divided into two groups; significant improvement or complete freedom from symptoms and slight or no improvement. The results (**Table 1**) show that the patients believed second generation antihistamines to be significantly (*P*<0.005) more effective than first generation drugs.

**Table 1:** Comparison of first and second generation H_1_-antihistamines in chronic spontaneous urticaria.

	First generation	Second generation	Significance of Difference
**Effectiveness:**			
Significant or total improvement	15.6%	26.8%	
Slight or no improvement	84.4%	73.2%	*P*<0.005
	(n = 147)	(n = 721)	
**Unwanted Effects:**			
Unwanted Effects	46.5%	31.6%	*P*<0.001
Sedation	38.9%	22.7%	*P*<0.001
	(n = 157)	(n = 699)	

The first generation ‘sedating’ antihistamines were clemastine, dimethindene, hydroxyzine and promethazine and the second generation, ‘non-sedating’ antihistamines were cetirizine, desloratadine, ebastine, fexofenadine, levocetirizine, loratadine, mizolastine and rupatadine. All were taken at the standard licensed dose. Statistical comparisons were made using Fisher's exact test. n indicates the number of reports received for each condition.

The patients were also asked whether or not taking a standard dose of H_1_-antihistamines caused unwanted effects. The responses (**Table 1**) showed first generation antihistamines to cause significantly (*P*<0.001) more unwanted effects than second generation drugs. Furthermore, more respondents stated that they felt tired when taking first generation antihistamines compared with second generation drugs. Again, the difference was highly significant (*P*<0.001).

### Up-dosing with H_1_-antihistamines

**Figure 3:**
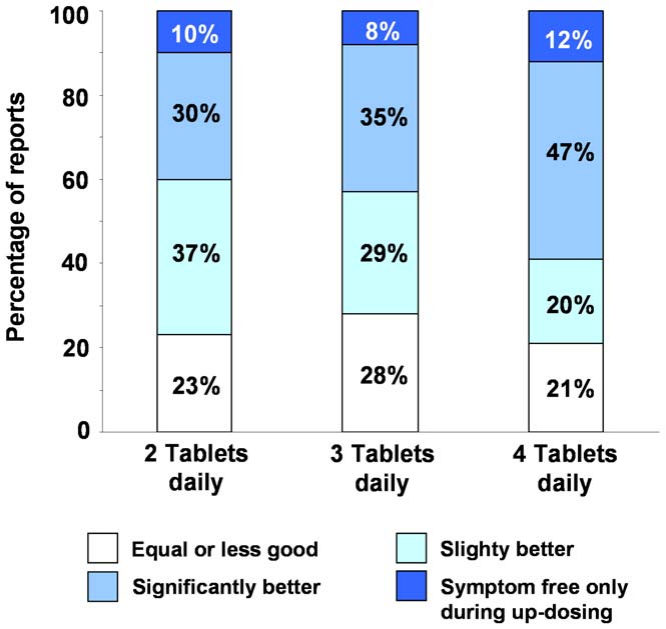
Effectiveness of H_1_-antihistamine updosing in chronic spontaneous urticaria. The reported effectiveness of up-dosing with second generation H_1_-antihistamines compared with standard dose therapy in chronic spontaneous urticaria. The number of patient reports in each group are; 2 tablets = 108, 3 tablets = 72 and 4 tablets = 85.

A total of 238 of the 319 patients (75%) in the survey stated that they had increased their daily dose of antihistamine to two, three or four tablets daily. Of these, 216 (68%) of the patients stated that they had taken this course of action because of the lack of effectiveness when taking a standard dose. **Figure 3** shows the effectiveness of up-dosing with second generation H_1_-antihistamines. Of the reports from individuals who had stated that they had up-dosed, 40%, 42% and 54% showed significant added benefit from taking 2, 3 or 4 tablets daily rather than standard dose therapy while approximately one quarter stated that no additional benefit had been derived.

### Unwanted effects and concerns with up-dosing

The number of reports of unwanted effects and sedation following up-dosing with second generation H_1_-antihistamines are shown in **Table 2** and are not statistically different from those reported for the use of standard doses of antihistamines. However, of the 135 reports about unwanted effects, 85 gave also information on the magnitude at high doses in comparison to regular doses of the same second generation H_1_-antihistamine: 30 (35%) stated that the unwanted effects were considerably worse and 33 (39%) stated that they were somewhat worse.

**Table 2:** Unwanted effects and sedation while using standard doses or up-dosing with H_1_-antihistamines in chronic spontaneous urticaria.

	Standard dosage	Up-dosing	Significance of Difference
**Unwanted Effects**	31.6%	32.5%	not significant(*P* = 0.791)
	(n = 699)	(n = 416)	
**Sedation**	22.7%	22.1%	not significant(*P* = 0.824)
	(n = 699)	(n = 416)	

The reports included in this table are from patients who had taken either the standard dose or two, three or four times the standard dose each day of a second generation H_1_-antihistamine. Statistical comparisons were made using Fisher's exact test. n indicates the number of reports received for each condition.

Patients also expressed concerns about up-dosing with H_1_-antihistamines. Of the 319 respondents, 26% were worried about possible side effects, 23% about possible long term harmful effects, 19% about the possible loss of efficacy, 9% about becoming dependent on their medication and 3% about other unspecified effects.

## Discussion

This survey shows that, from a patients' perspective, a standard licensed dose of an H_1_-antihistamine often fails to provide adequate symptomatic relief in chronic spontaneous urticaria. This is evidenced by the reported 322 changes of medication and the finding that 75% of the respondents reported to increasing their daily dose of antihistamines in order to improve symptomatic control.

Because this survey asked questions relating to treatment over many years, many patients stated that they either had been or are being treated with first generation H_1_-antihistamines. These drugs, many of which have been available for more than 50 years, have pronounced anticholinergic effects and central nervous system sedative actions which limit the doses at which they can be given [17], [18]. Consequently, in clinical usage for urticaria, first generation H_1_-antihistamines are generally less effective than second generation drugs [19], a conclusion supported by our survey. Furthermore, it is well established that taking first generation H_1_-antihistamines in standard doses frequently leads to side effects of which daytime somnolence, sedation, drowsiness, fatigue and impaired concentration and memory are the most prominent [17], [20], [21]. The finding in this survey that first generation H_1_-antihistamines were associated with significantly more side effects and sedation than second generation drugs supports these conclusions. These results support the recommendation in the guidelines [8] that the first line treatment for chronic urticaria should be second generation H_1_-antihistamines rather than the old sedating first generation H_1_-antihistamines.

Of the responses from patients taking second generation antihistamines, 23% stated that the feeling of sedation was a problem. This is considerably higher than somnolence levels cited in clinical studies [20]–[22]. However, the majority of sedation studies with H_1_-antihistamines are performed in either healthy individuals or individuals with mild disease rather than in conditions, such as severe chronic urticaria, in which sleep disturbances are a major issue [23]. Thus, in surveys such as ours, it is difficult to discern what proportion of the ‘sedation’ reported by the patients is due to the drug and what is due to their condition.

Of the reports from individuals who had stated that they had up-dosed their H_1_-antihistamine, approximately half stated that there was a significant benefit compared with standard dose therapy while approximately one quarter stated that no additional benefit had been derived. This finding that in many cases of chronic spontaneous urticaria, therapy with H_1_-antihistamines is not sufficient, even at higher than licensed doses, confirms previous studies [2], [11], [16].

Perhaps the major concern shared by both patients and doctors concerns the possibility of somnolence with up-dosing of H_1_-antihistamines. However, in this survey, the percentage of reports of sedation following up-dosing compared with standard doses of H_1_-antihistamines was not statistically different. There are two possible reasons for this. The first is that the relief from urticaria-related discomfort led to a better quality of sleep with subsequent less sedation during the day [16]. The second possibility, which is likely to occur in parallel, is the development of tolerance to the central nervous sedative effects of the antihistamines which occurs with prolonged periods of administration [24], [25].

However, of the reports received following antihistamine up-dosing, many stated that unwanted effects were worse than with the standard dose. This illustrates the patient variability to the central nervous effects of second generation H_1_-antihistamines and that the blanket term of ‘non-sedating’ for this class of drug is not appropriate for many patients.

### Strengths and limitations of this study

The major strengths of this study are: first, that it canvassed the opinions of patients from many walks of life from all over Germany; second, patients were only admitted after a physician had confirmed the diagnosis of chronic spontaneous urticaria; many patients had taken both first and second generation antihistamines and were, therefore, able to compare their efficacy and unwanted effects; and, fourth, three quarters of the patients had up-dosed with antihistamines. The weaknesses of the study are: first, many patients commented on drugs that they had taken many years ago and, therefore, their memory of relevant details may be a little unreliable (recall bias); second, because of the size of the cohort, co-morbidities could not be included in the analysis; and, third, due to the way of patient recruitment, a selection effect can not be excluded.

### Conclusions

In conclusion, this survey has shown that approximately three quarters of reports indicate that patients obtain additional benefit from up-dosing with H_1_-antihistamines. Also, in approximately three quarters of responses, the reported frequency of side effects was no greater than with standard doses. Furthermore, the survey shows a clear benefit of second generation over first generation drugs in both efficacy and their side effects. These results, therefore, support the guidelines recommendations [8] that the first line treatment for chronic spontaneous urticaria should be second generation H_1_-antihistamines and that, if standard dosing is not effective, the dosage should be increased up to four-fold.

## References

[1] Zuberbier T, Asero R, Bindslev-Jensen C, Walter CG, Church MK (2009). EAACI/GA^2^LEN/EDF/WAO guideline: definition, classification and diagnosis of urticaria.. Allergy.

[2] Maurer M, Weller K, Bindslev-Jensen C, Gimenez-Arnau A, Bousquet PJ (2011). Unmet clinical needs in chronic spontaneous urticaria. A GA^2^LEN task force report.. Allergy.

[3] van der Valk PG, Moret G, Kiemeney LA (2002). The natural history of chronic urticaria and angioedema in patients visiting a tertiary referral centre.. Br J Dermatol.

[4] Toubi E, Kessel A, Avshovich N, Bamberger E, Sabo E (2004). Clinical and laboratory parameters in predicting chronic urticaria duration: a prospective study of 139 patients.. Allergy.

[5] O'Donnell BF, Lawlor F, Simpson J, Morgan M, Greaves MW (1997). The impact of chronic urticaria on the quality of life.. Br J Dermatol.

[6] Poon E, Seed PT, Greaves MW, Kobza-Black A (1999). The extent and nature of disability in different urticarial conditions.. Br J Dermatol.

[7] Grob JJ, Revuz J, Ortonne JP, Auquier P, Lorette G (2005). Comparative study of the impact of chronic urticaria, psoriasis and atopic dermatitis on the quality of life.. Br J Dermatol.

[8] Zuberbier T, Asero R, Bindslev-Jensen C, Walter CG, Church MK (2009). EAACI/GA^2^LEN/EDF/WAO guideline: management of urticaria.. Allergy.

[9] Zuberbier T, Munzberger C, Haustein U, Trippas E, Burtin B (1996). Double-blind crossover study of high-dose cetirizine in cholinergic urticaria.. Dermatology.

[10] Kameyoshi Y, Tanaka T, Mihara S, Takahagi S, Niimi N (2007). Increasing the dose of cetirizine may lead to better control of chronic idiopathic urticaria: an open study of 21 patients.. Br J Dermatol.

[11] Gimenez-Arnau A, Izquierdo I, Maurer M (2009). The use of a responder analysis to identify clinically meaningful differences in chronic urticaria patients following placebo- controlled treatment with rupatadine 10 and 20 mg.. J Eur Acad Dermatol Venereol.

[12] Metz M, Scholz E, Ferran M, Izquierdo I, Gimenez-Arnau A (2010). Rupatadine and its effects on symptom control, stimulation time, and temperature thresholds in patients with acquired cold urticaria.. Ann Allergy Asthma Immunol.

[13] Asero R (2007). Chronic unremitting urticaria: is the use of antihistamines above the licensed dose effective? A preliminary study of cetirizine at licensed and above-licensed doses.. Clin Exp Dermatol.

[14] Godse KV, Nadkarni NJ, Jani G, Ghate S (2010). Fexofenadine in higher doses in chronic spontaneous urticaria.. Indian Dermatol Online J.

[15] Siebenhaar F, Degener F, Zuberbier T, Martus P, Maurer M (2009). High-dose desloratadine decreases wheal volume and improves cold provocation thresholds compared with standard-dose treatment in patients with acquired cold urticaria: a randomized, placebo-controlled, crossover study.. J Allergy Clin Immunol.

[16] Staevska M, Popov TA, Kralimarkova T, Lazarova C, Kraeva S (2010). The effectiveness of levocetirizine and desloratadine in up to 4 times conventional doses in difficult-to-treat urticaria.. J Allergy Clin Immunol.

[17] Simons FE (1994). H_1_-receptor antagonists. Comparative tolerability and safety.. Drug Saf.

[18] Church MK, Maurer M, Simons FE, Bindslev-Jensen C, van Cauwenberge P (2010). Risk of first-generation H_1_-antihistamines: a GA^2^LEN position paper.. Allergy.

[19] Simons FE, Silver NA, Gu X, Simons KJ (2002). Clinical pharmacology of H_1_-antihistamines in the skin.. J Allergy Clin Immunol.

[20] Simons FE (1999). H_1_-receptor antagonists: safety issues.. Ann Allergy Asthma Immunol.

[21] Simons FE (2004). Advances in H1-antihistamines.. N Engl J Med.

[22] Devillier P, Roche N, Faisy C (2008). Clinical pharmacokinetics and pharmacodynamics of desloratadine, fexofenadine and levocetirizine : a comparative review.. Clin Pharmacokinet.

[23] Maurer M, Ortonne JP, Zuberbier T (2009). Chronic urticaria: an internet survey of health behaviours, symptom patterns and treatment needs in European adult patients.. Br J Dermatol.

[24] Richardson GS, Roehrs TA, Rosenthal L, Koshorek G, Roth T (2002). Tolerance to daytime sedative effects of H1 antihistamines.. J Clin Psychopharmacol.

[25] Verster JC, Volkerts ER (2004). Antihistamines and driving ability: evidence from on-the-road driving studies during normal traffic.. Ann Allergy Asthma Immunol.

